# Structural basis of *Fp*GalNase and its combination with *Fp*GalNAcDeAc for efficient A-to-O blood group conversion

**DOI:** 10.1186/s40164-025-00599-7

**Published:** 2025-01-24

**Authors:** Meiling Zhou, Kaishan Luo, Chao Su, Yan Sun, Zuyan Huang, Shuo Ma, Xun Gao, Jiwei Wang, Chen Zhang, Pengcheng Han, Guoqiu Wu

**Affiliations:** Jiangsu Provincial Key Laboratory of Critical Care Medicine, Advanced Institute for Life and Health, Center of Clinical Laboratory Medicine, Department of Pharmacy, School of Medicine, Zhongda Hospital, Southeast University, Nanjing, 210009 China

**Keywords:** Blood transfusion, *Fp*GalNAcDeAc, *Fp*GalNase, Galactosaminidase, N-acetylgalactosamine deacetylase, cryo-EM structure, Blood group conversion

## Abstract

Transfusion safety and blood typing continue to present significant challenges in clinical practice, including risks of incorrect blood transfusions and blood shortages. One promising solution is the enzymatic conversion of all red blood cell (RBC) types into universal O-type RBCs. However, the major obstacle to this strategy is the relatively low catalytic efficiency of the enzymes involved. In this study, we investigated two enzymes from *Flavonifractor plautii*, N-acetylgalactosamine deacetylase (*Fp*GalNAcDeAc) and galactosaminidase (*Fp*GalNase), which demonstrate synergistic activity in efficiently converting A-type RBCs to O-type. We optimized treatment conditions, achieving over 99% conversion in just five minutes using phosphate buffer saline and a 16 nM enzyme concentration. Additionally, we engineered two fusion proteins, *Fp*GalNAcDeAc-*Fp*GalNase and *Fp*GalNase-*Fp*GalNAcDeAc, which showed a 28-fold increase in catalytic efficiency compared to the enzyme mixture. Using cryo-electron microscopy, we resolved the full-length structure of *Fp*GalNase, identifying critical active site residues involved in its catalytic mechanism. This study provides essential structural and biochemical insights for clinical applications in blood group conversion, offering a promising approach for producing universal O-type RBCs.

**Supplementary Information:**

The online version contains supplementary material available at 10.1186/s40164-025-00599-7.

**To the editor:**

Worldwide, over 118 million blood donations are needed each year [1], highlighting an ongoing demand for O-type red blood cells (RBCs) due to their universal compatibility. O-type RBCs lack immunodominant sugars found in A (α-N-acetylgalactosamine) and B antigen (galactose) [2, 3], making them essential in emergencies and when matching blood types is not feasible [4]. While antigen-sheltering and gene knockout methods pose significant safety and ethical concerns, enzymatic conversion of A- or B-type RBCs to O-type represents a promising solution. However, current enzymatic methods have limited clinical application due to low catalytic efficiency [5–8], prompting a search for more effective solutions.

Recent studies identified two synergistic enzymes from *Flavonifractor plautii*, namely, N-acetylgalactosamine deacetylase (*Fp*GalNAcDeAc) and galactosaminidase (*Fp*GalNase), which efficiently convert A-type RBCs to O-type by removing A antigens [8] (Fig. S1). Here, we provide optimized protocols and structural insights for *Fp*GalNase, advancing the potential for clinical application in A-to-O conversion.

We isolated *Flavonifractor plautii* from patient samples cultured on Columbia blood agar plates (Fig. S2A). Sequencing analysis of *Fp*GalNAcDeAc and *Fp*GalNase revealed three mutations in each protein, including V284A in *Fp*GalNAcDeAc and P566L in *Fp*GalNase, located within their catalytic domains (Fig. 1A). Both enzymes were purified (Fig. 1A) and successfully converted A-type RBCs to O-type in samples from healthy donors, without affecting B-type, AB-type, or O-type RBCs when treated with either enzyme alone (Fig. S2B). Microscopic analysis showed that A-type RBCs co-incubated with both enzymes did not agglutinate with anti-A antibodies but did with anti-H antibodies, maintaining their typical biconcave morphology and size throughout the enzyme treatment (Fig. S2C-S2E), indicating treatment safety.

**Fig. 1:**
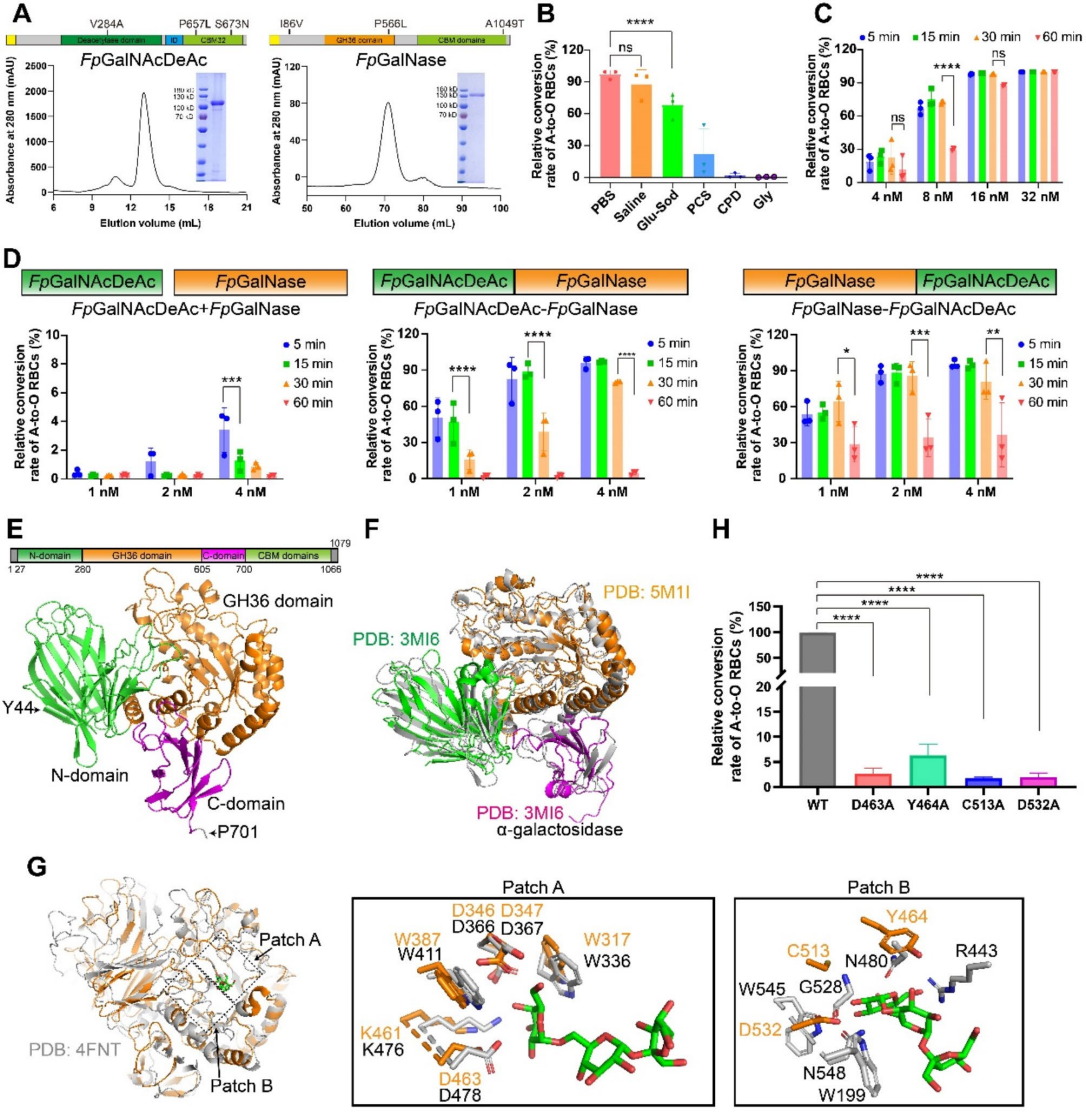
Optimized and enhanced enzymatic activity of *Fp*GalNAcDeAc and *Fp*GalNase, and structural characterization of *Fp*GalNase. (**A**) Analytical gel filtration of *Fp*GalNAcDeAc (left) and *Fp*GalNase (right) protein. The 280-nm absorbance curve from Superdex 200 10/300 GL or Hiload 16/60 superdex 200 10/300 PG column and the SDS-PAGE migration profile are shown. Mutation sites in *Fp*GalNAcDeAc and *Fp*GalNase are indicated, based on reference sequences from the NCBI database (accession codes P0DTR4 and P0DTR5, respectively). CBM: carbohydrate-binding motif. (**B**-**D**) Statistical plots of FACS results display the percentage of A-to-O conversion in various conditions: across different buffers (B), at varying incubation time and enzyme concentrations (C), and with different enzyme combinations (D), including *Fp*GalNAcDeAc and *Fp*GalNase mixture (left), fusion protein *Fp*GalNAcDeAc-*Fp*GalNase (middle), and fusion protein *Fp*GalNase-*Fp*GalNAcDeAc (right). All data are shown as means ± SD from three independent experiments. Statistical significance is indicated (ns: no significance, *p < 0.05, **p < 0.01, ***p < 0.001, ****p < 0.0001). (**E**) Schematic and cartoon representations of the *Fp*GalNase structure, highlighting the N-domain (green), GH36 domain (orange), and C-domain (magenta). (**F**) Structural alignment between the N-, GH36, and C-domains of *Fp*GalNase (gray) and their respective structurally similar domains. (**G**) Structural comparison of *Fp*GalNase with α-galactosidase (PDB: 4FNT), showing the full alignment (left) and detailed interactions in active site region patch A (middle) and patch B (right). Residues in *Fp*GalNase and GH36 α-galactosidase are colored in orange and gray, respectively. (**H**) A-to-O conversion rates of A-type RBCs treated with the *Fp*GalNAcDeAc and either *Fp*GalNase or *Fp*GalNase mutants. Data are shown as means ± SD from three independent experiments. Statistical significance is indicated (****p < 0.0001)

To optimize enzyme activity, we conducted fluorescence-activated cell sorting (FACS) assays across various buffers, including saline, phosphate-buffered saline (PBS), glucose sodium chloride solution, glycine buffer, phosphate citrate saline buffer, and citrate phosphate dextrose, along with varying enzyme concentrations (4 nM, 8 nM, 16 nM, and 32 nM) and incubation times (5, 15, 30, and 60 min). Among the tested buffers, PBS and saline facilitated the highest A-to-O conversion rates (Fig. 1B and S3A). The additional components in the other buffers compared to PBS may have influenced catalytic efficiency. Notably, co-incubating *Fp*GalNAcDeAc and *Fp*GalNase with RBCs in PBS at concentrations of 16 nM or 32 nM for just 5 min resulted in over 99% A-to-O conversion, significantly higher than that achieved with 4 nM or 8 nM (Fig. 1C and S3B). To maximize both economic and catalytic efficiency in clinical applications, the 16 nM concentration should be prioritized. However, extending the incubation time from 30 to 60 min, and using lower enzyme concentrations (4 nM and 8 nM), led to significant reductions in conversion efficiency due to reversible reactions (Fig. 1C and S3B), highlighting the importance of maintaining optimized conditions for clinical applications of these enzymes.

To further improve enzymatic activity, we engineered two covalently linked fusion proteins: *Fp*GalNAcDeAc-*Fp*GalNase and *Fp*GalNase-*Fp*GalNAcDeAc. These fusion proteins exhibited substantially higher catalytic activity than the two enzymes used separately, achieving ~ 96% conversion at only 4 nM enzyme concentration with a 5-minute incubation (Fig. 1D and S4). This represents a 28-fold increase in efficiency compared to the non-fused enzyme pair (Fig. 1D and S4). After storage at -80 °C for five months, the fusion protein retained similar conversion efficiency (Fig. S5). This enhancement is likely due to substrate channeling facilitated by C-terminal carbohydrate-binding motif (CBM), which enables the product of one enzyme to be directly transferred to the active site of the second enzyme, bypassing diffusion through the bulk solution [9].

Understanding enzyme structure is essential for enhancing catalytic efficiency. While the structure of *Fp*GalNAcDeAc has been resolved [8], elucidating the catalytic mechanism of *Fp*GalNase is crucial. We successfully determined the cryo-electron microscopy (cryo-EM) structure of full-length *Fp*GalNase at 2.56 Å resolution (Fig. 1E). The N-terminal region (residue 27–699) adopts a disc-like conformation divided into three segments: the N-domain, the central GH36 domain, and the C-domain (Fig. 1E). Structural similarity analysis using the Dali tool [10] revealed that each domain exhibits a structure similar to α-galactosidase (Fig. 1F), despite low sequence identity.

To identify key catalytic residues, we compared the GH36 structure of *Fp*GalNase with that of α-galactosidase complexed with raffinose (PDB: 4FNT). Conserved residues in *Fp*GalNase active site include W317, D346, D347, W387, K461, and D463 (patch A), along with specific non-conserved residues (Y464, C513, and D532) in patch B, which afford a more open catalytic center conformation (Fig. 1G) and may determine its substrate specificity. Site-directed mutagenesis of D463A, Y464A, C513A, and D532A confirmed the functional importance of these residues, as mutations led to significant reductions in conversion activity, with D463A, C513A, and D532A nearly inactive (Fig. 1H and S6A), and the mutants were unable to convert A-type RBCs to O-type in samples from healthy donors (Fig. S6B). Moreover, comparison between the experimental structure and the AlphaFold-predicted structure revealed a significant deviation in the region from D559 to W564 in the catalytic pocket (Fig. S7), highlighting the critical role of experimental data in refining structural models.

In conclusion, we optimized conditions for *Fp*GalNAcDeAc and *Fp*GalNase in A-to-O blood group conversion and demonstrated that their fusion protein improves catalytic efficiency. The resolved structures of *Fp*GalNase revealed unique active centers. These findings are critical for the clinical application of A-to-O conversion, with significant implications for producing universal O-type RBCs for transfusion and disease-associated research.

## Electronic supplementary material

Below is the link to the electronic supplementary material.

Supplementary Material 1

## Data Availability

The cryo‐EM density maps and corresponding atomic coordinates have been deposited in the Electron Microscopy Data Bank (EMDB) and Protein Data Bank (PDB), respectively. The accession numbers for the FpGalNase cryo‐EM structure in EMDB and PDB are EMD-37989 and 8 × 1B, respectively.

## Abbreviations

RBCs: Red blood cells
FpGalNAcDeAc: Flavonifractor plautii N-acetylgalactosamine deacetylase
FpGalNase: Flavonifractor plautii galactosaminidase
cryo-EM: cryo-electron microscopy
FACS: Fluorescence-activated cell sorting
Glu-Sod: Glucose sodium chloride solution
PBS: Phosphate buffer saline
Gly: Glycine
PCS: Phosphate citrate saline
CPD: Citrate phosphate dextrose
CBM: Carbohydrate-binding motif
EMDB: Electron microscopy data bank
PDB: Protein data bank
IPTG: Isopropyl-β-D-thiogalactoside
CTF: Contrast transfer function
SD: Standard deviation
