# Supplementary material for: Structural basis of FpGalNase and its combination with FpGalNAcDeAc for efficient A-to-O blood group conversion
Source: Exp Hematol Oncol. 2025 Jan 24;14:7. doi: 10.1186/s40164-025-00599-7 (PMC11762096; doi:10.1186/s40164-025-00599-7)
Supplement: Supplementary file 1 — Supplementary Material 1 [file 40164_2025_599_MOESM1_ESM.docx]

**Supplementary information**

**Structural basis of *Fp*GalNase and its combination with *Fp*GalNAcDeAc for efficient A-to-O blood group conversion**

Meiling Zhou^1,#^, Kaishan Luo^1,#^, Chao Su^1,#^, Yan Sun^1^, Zuyan Huang^1^, Shuo Ma^1^, Xun Gao^1^, Jiwei Wang^1^, Chen Zhang^1^, Pengcheng Han^1,*^, Guoqiu Wu^1,*^

**Methods**

**Strain** **isolation**

A strain of anaerobic bacteria was isolated from the blood sample of a patient with an AB-type, Rh-positive bloodstream infection, admitted to Zhongda Hospital. The isolate was cultured and amplified on Columbia blood agar plates under strict anaerobic conditions. Gram staining was performed, and microscopy was used to assess the bacterial morphology and characteristics. The species of the bacterium was identified using MALDI-TOF MS.

**Polymerase chain reaction (PCR)**

The bacterial genomic DNA was extracted from the isolated *Flavonifractor plautii* strain. Based on the gene sequences of *Fp*GalNAcDeAc (P0DTR4) and *Fp*GalNase (P0DTR5) available in the NCBI database, we designed specific primers for PCR amplification. The primer sequences were as follows: *Fp*GalNAcDeAc forward primer: 5'-ATGGTCTCGCCATGCAGACTCCAGCGAGTCCG-3', reverse primer: 5'-ATGGTCTCGATTCTTATTCTCCCACATACGAAAAATAGTCG-3'. *Fp*GalNase forward primer: 5'-ATGGTCTCGCCATCGTGGTAAAAAGTTCATATCACTCAC-3', reverse primer: 5'-ATGGTCTCGATTCTTATGCGTTAGTGGTATAAGTCAAATAGTC-3'. Using the extracted genomic DNA as the template, PCR amplification was performed with the following conditions: denaturation at 98 ℃ for 3 minutes, annealing at 65 ℃ for 30 s, extension at 72 ℃ for 1 minute, for a total of 30 cycles. After amplification and purification, the PCR products were sequenced.

**Gene cloning, protein expression, and purification**

The DNA sequences of *Fp*GalNAcDeAc and *Fp*GalNase, each containing a C-terminal hexa-His tag, were inserted into pET-25b vector *via* 5' *Nco*I and 3' *Xho*I. The coding sequences of *Fp*GalNAcDeAc-*Fp*GalNase or *Fp*GalNAcDeAc-*Fp*GalNase were inserted into a pET-21a vector with a C-terminal hexa-His tag sequence. The *Fp*GalNase mutants, including D463A, Y464A, C513A, and D532A, were constructed *via* site-directed mutagenesis. The recombinant plasmids were then transformed into *Escherichia coli* BL21(DE3) cells (Invitrogen, USA). The transformed cells were cultured in 500 mL of LB medium containing 50 μg/mL Ampicillin. Protein expression was induced by adding 0.5 mM isopropyl-β-D-thiogalactoside (IPTG), and the cultures were incubated overnight at 25 ℃. After induction, the bacterial cells were harvested by centrifugation and subjected to lysis *via* ultrasonication. The lysates were centrifuged to remove cellular debris, and the supernatants were filtered. The target proteins were purified using a HisTrap HP 5 mL affinity column (Cytiva), with the proteins eluted in an elution buffer composed of 20 mM Tris (pH 8.0), 150 mM NaCl, and 300 mM imidazole. Further purification of the target proteins was performed *via* gel-filtration chromatography using a HiLoad 16/600 superdex 200 PG column or Superdex 200 10/300 GL (Cytiva) in a PBS buffer. The purified proteins were then analyzed for further experimentation.

**Enzymatic treatment of RBCs**

5 mL of commercially available standard A-type RBCs (0.8 %) were centrifuged at 1000 g for 5 minutes and washed twice with a specific buffer solution. The enzymes *Fp*GalNAcDeAc and *Fp*GalNase or its mutants were then mixed with the RBCs in 100 μL of buffer, adjusting the hematocrit to 40 %. The cells were gently shaken every 10 minutes during the enzymatic reaction. After the reaction, the RBCs were washed twice with a PBS buffer to prepare them for further experiments.

**RBCs morphology**

5 μL of pre- and post-enzymatic RBCs mixed with the corresponding antibodies (anti-A and anti-H) were carefully dropped onto a microscope slide. The sample was observed under a Leica DM4M microscope (Germany) within 5 minutes to capture any agglutination or other interactions between the RBCs and antibodies. Photographs of the interactions were taken immediately to document the results for further analysis.

**Atomic force microscopy**

5 μL of pre- and post-enzymatic A-type RBC samples were carefully added dropwise onto a freshly cleaved mica sheet to ensure an even spread of the cells. The mica sheet was then dried using pure nitrogen gas to remove excess liquid and prevent distortion of the RBC morphology. Once dry, the mica sheet was secured onto the sample stage of the atomic force microscope (Dimension ICON, Bruker, USA) to ensure flatness and stability for accurate imaging. Atomic force microscopy was subsequently performed to analyze the surface characteristics of the RBCs.

**Scanning electron microscopy**

5 μL pre- and post-enzymatic A-type RBCs samples were used for imaging by biological scanning electron microscopy (Regulus8100, HITACHI, Japan). Pre-cooled 2.5 % glutaraldehyde was carefully added to the RBCs and the samples were incubated at 4 ℃ overnight for fixation. The samples were then washed three times with PBS and subsequently fixed with 1% osmium solution for 1 hour, following by three additional PBS washes. The samples were then washed with increasing concentrations of ethanol, including 50 %, 60 %, 70 %, 80 %, and 90 %, for 15 minutes at each concentration. Afterward, the RBCs were dehydrated using graded concentrations of ethanol (90 %, and 95 %), also for 15 minutes at each step. Finally, the samples were treated with 100 % acetone twice, for 15 minutes each. Following acetone treatment, the samples were dried using CO₂ for further electron microscopy analysis

**RBC blood group conversion of clinical samples**

The study was approved by the Medical Ethics Committee of Zhongda Hospital, affiliated with Southeast University (2024ZDSYLL299-P01), and all procedures adhered to their ethical guidelines. Written informed consent was obtained from all subjects. Leukocyte-free RBCs from healthy donors, collected within 5 days, were supplied by the Transfusion Department of Zhongda Hospital. RBC samples were obtained from 20 clinically healthy human subjects with A-, B-, AB-, and O-type blood. For each sample, 200 μL of RBCs were centrifuged at 1000 g for 5 min and washed twice with saline. The 200 μL 40 % RBCs were then incubated with *Fp*GalNase or its mutants and *Fp*GalNAcDeAc enzymes (32 nM) in buffer at 26 ℃ for 30 minutes. Following the enzymatic reaction, the RBCs were washed twice with saline and resuspended as a 0.8 % saline suspension.

For blood group antigen testing, 50 μL of RBCs suspension was added into microtubes of an ABO blood group antigen test card. Samples were centrifuged for 5 minutes (900 rpm for 2 minutes, 1500 rpm for 3 minutes) using a cassette-type special centrifuge (TD-3A, ChangChun Boyan Scientific Instrument Co., Ltd, China). A positive reaction was indicated by the formation of a specific antigen-antibody complex within the microcolumn gel. Hemolysis was marked by a clear, transparent red color in the wells of the gel, while biclustering (mixed agglutination) was characterized by agglutinated red blood cells remaining at the top of the gel, and non-agglutinated cells sinking to the bottom of the microcolumn.

**Optimization for buffers**

To determine the optimal buffer system for enzyme incubation with RBCs, several buffer solutions were tested by co-incubating A-type RBCs with 16 nM *Fp*GalNAcDeAc and *Fp*GalNase in 100 μL of different buffer solutions. The buffers used were saline, Gly buffer (250 mM glycine, 3 mM NaCl, pH 6.8), PCS (77.25 mM Na_2_HPO_4_, 11.375 mM citric acid, 53.9 mM NaCl, pH 6.8), CPD buffer (10.2 mM sodium citrate, 7.4 mM dihydrogen phosphate potassium, 111 mM dextrose), 5 % Glu-Sod at room temperature (26 ℃) for 15 minutes. Following incubation, the samples were centrifuged at 1000 g for 5 minutes to pellet the RBCs. The cells were then prepared for FACS analysis to assess the effect of each buffer on the enzymatic conversion process.

**Optimization for concentration and time**

Different working concentrations of *Fp*GalNAcDeAc and *Fp*GalNase (4 nM, 8 nM, 16 nM, and 32 nM) were co-incubated with A-type RBCs in PBS solution for various time intervals (5 minutes, 15 minutes, 30 minutes, and 60 minutes). After the designated incubation periods, the cells were collected and washed to remove excess enzymes. The treated RBCs were then prepared for FACS analysis to evaluate the extent of enzymatic conversion at each concentration and time point.

**FACS**

The enzymes were incubated with A-type RBCs at different conditions. Following incubation, the proportions of A and H antigens on the RBCs surface were analyzed *via* FACS. Briefly, 5 μL of enzyme-treated RBCs were washed twice with PBS, resuspended in 200 μL PBS, and incubated with a 1:100 dilution of APC-conjugated anti-A antibody (Alexa Fluor 647 mouse anti-human blood group A, BD Pharmingen, 565384) at 26 ℃ for 30 minutes to detect residual A antigen. Simultaneously, a 1:10 dilution of FITC-conjugated Ulex europaeus agglutinin I (FITC-UEA-I, Sigma, I9006) was used to detect H antigen expression on the RBC surface. Flow cytometry was performed using a BD LSRFortessa (USA). All incubations were conducted in light-protected conditions to minimize photo-damage.

**Cryo-EM sample preparation and data collection**

To prepare cryo-EM sample, 4.0 µL purified *Fp*GalNase protein in PBS buffer were applied to both 1.2/1.3 300 mesh GO(Au) Quantifoil grid and 60 s glow-discharged 300 mesh NiTi foli grid (Au 1.2/1.3). The protein concentration used were 0.1 mg/mL and 0.3 mg/mL, respectively. Subsequently, the grids were blotted with filter paper for 2.5 s, using blotting force of -9 and -5, respectively, at 4 ℃ in 100 % humidity. They were then plunge-frozen in liquid ethane using a Vitrobot Mark IV (Thermo Fisher Scientific). The prepared grids were later transferred to a 300 kV Titan Krios transmission electron microscope equipped with Gatan K3 detector and GIF Quantum energy filter. Movies were collected at 105,000× magnification with a calibrated pixel size of 0.85 Å over a defocus range of -1.0 μm to -2.0 μm in super resolution counting mode, with a total dose of 50 e^-^/Å^2^ using EPU automated acquisition software.

**Image processing**

A total of 16,944 raw movies were motion corrected using MotionCor2 v1.2.4 [1]. Micrograph contrast transfer function (CTF) correction parameters were estimated using CTFFIND 4.0 [2] implemented in cryoSPARC v.4.1.2 [3]. Using blob picker, 5,229,387 particles were selected from 2,062 micrographs. After multiple rounds of 2D classification, the best class averages are used for Ab-initio reconstruction and further heterogeneous refinement. Topazwas used to train multiple models, and particles with weaker orientations were selected from the micrographs. After several rounds of two-dimensional classification, 205,498 particles were chosen for non-uniform refinement, resulting in a preliminary model with a resolution of 2.79 Å. From the remaining 114,342 images (including 7,270 images of the GO (Au) grid), the trained Topaz Model was used for particle picking, and the particles with weak orientation were selected. After multiple rounds of two-dimensional classification, 673,711 particles were finally selected for non-uniform refinement, yielding an electron density map with a resolution of 2.59 Å. The image processing workflow is summarized in Fig. S8. Details of overall resolution according to the gold standard FSC was provided in Table S1.

**Model building and structure refinement**

The Alphafold prediction model (P0DTR5) was fitted into the map using UCSF ChimeraX [4]. Mutation and manual adjustment were carried out with COOT v.0.9.3 [5]. Structural refinement was performed in Phenix [6]. Statistics associated with data collection, 3D reconstruction and model building were summarized in Table S1.

**Statistical analysis**

All values are expressed as mean ± standard deviation (SD). Statistical significance was analyzed using Tukey's multiple comparisons test for multiple groups in GraphPad Prism 8.0. p< 0.05 was considered statistically significant.

**Figure legends**


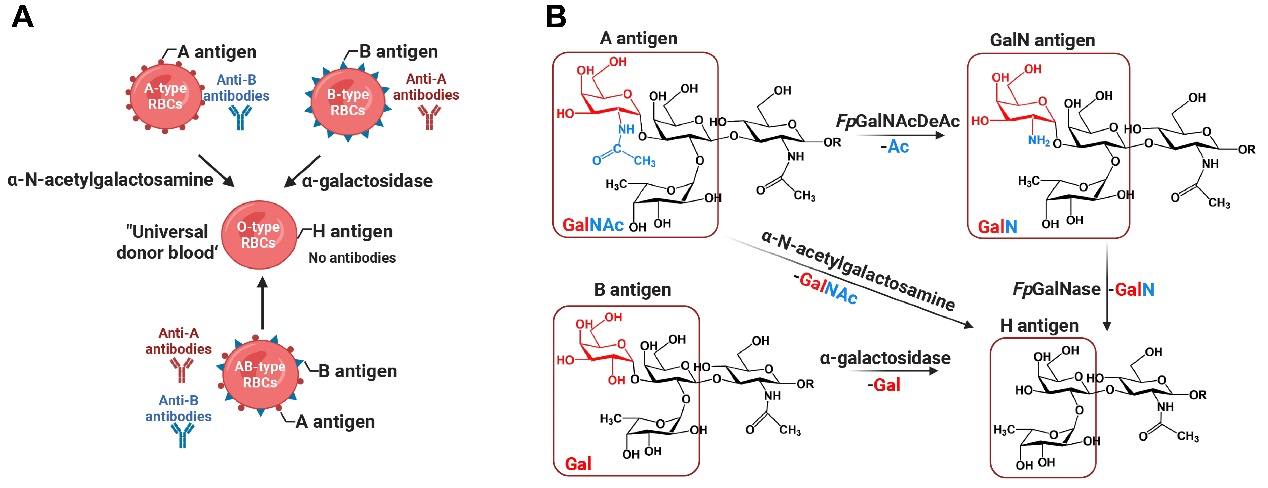


**Fig. S1** **Overview of A, B and H antigens on RBCs.** (**A**) Schematic representation of the enzymatic removal of ABO antigens from the surface of RBCs and the corresponding antibodies for each blood type. (**B**) Depiction of the specific enzymatic cleavage of terminal α-N-acetylgalactosamine (A antigen) or α-galactose (B antigen) monosaccharides, resulting in the conversion of A- or B-type RBCs to O-type RBCs.


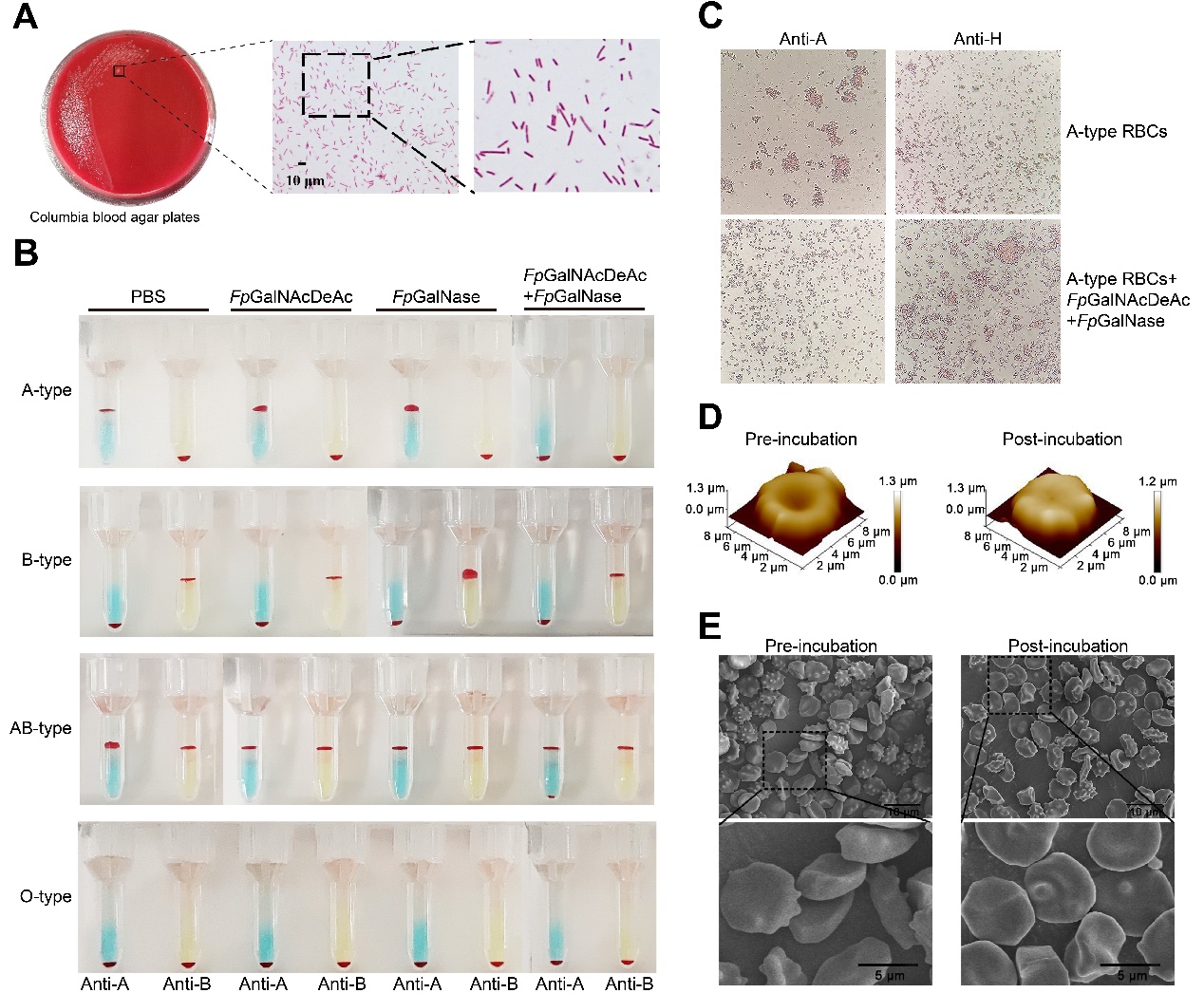


**Fig. S2 Conversed efficiency and morphology of A-type RBCs pre- and post-incubation with enzymes.** (**A**) *Flavonifractor plautii* bacteria were grown on Columbia blood agar plates in an anaerobic environment and observed microscopically. (**B**) Blood group identification of RBCs post-incubation with *Fp*GalNAcDeAc and *Fp*GalNase enzymes using a blood group identification card. (**C**) Microscopic images showing the agglutination reaction of anti-A or anti-H antibodies for A-type RBCs pre- and post-incubation with *Fp*GalNAcDeAc and *Fp*GalNase enzymes. (**D** and **E**) Atomic force microscopy (D) and scanning electron microscopy (E) images of A-type RBCs pre- and post-incubation with enzymes.


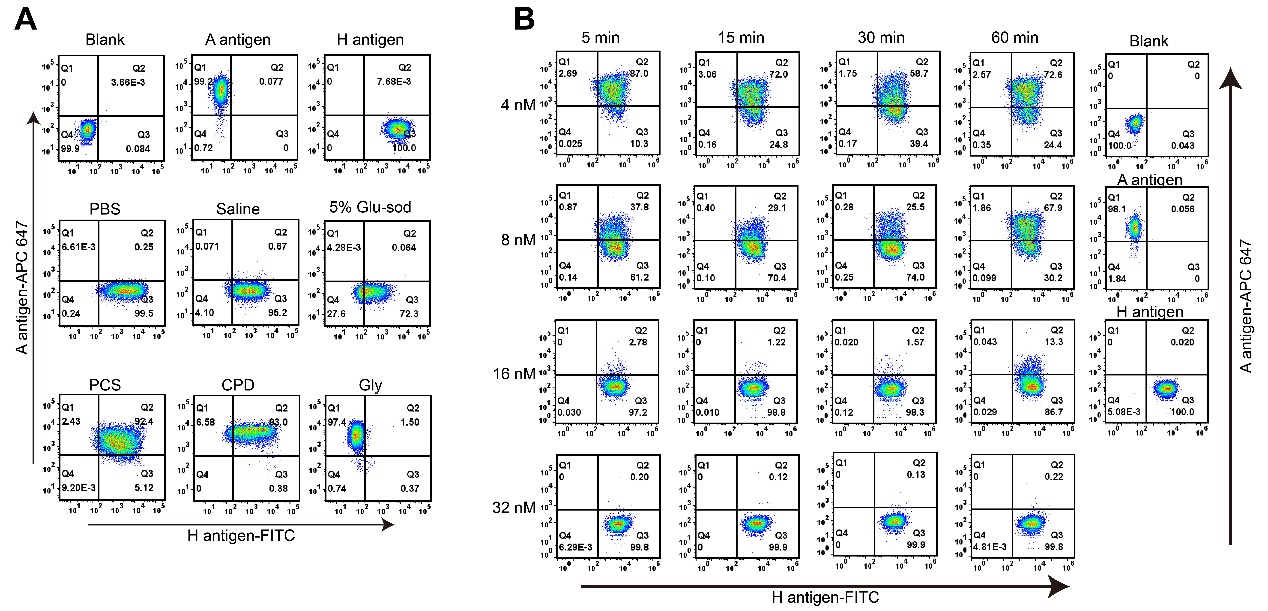


**Fig. S3** FACS analysis showing the relative amounts of A antigen and H antigen on the surface of A-type RBCs following incubation with *Fp*GalNAcDeAc and *Fp*GalNase in different buffers (A) or different incubation time and enzyme concentrations (B). Representative results from three independent experiments are shown.


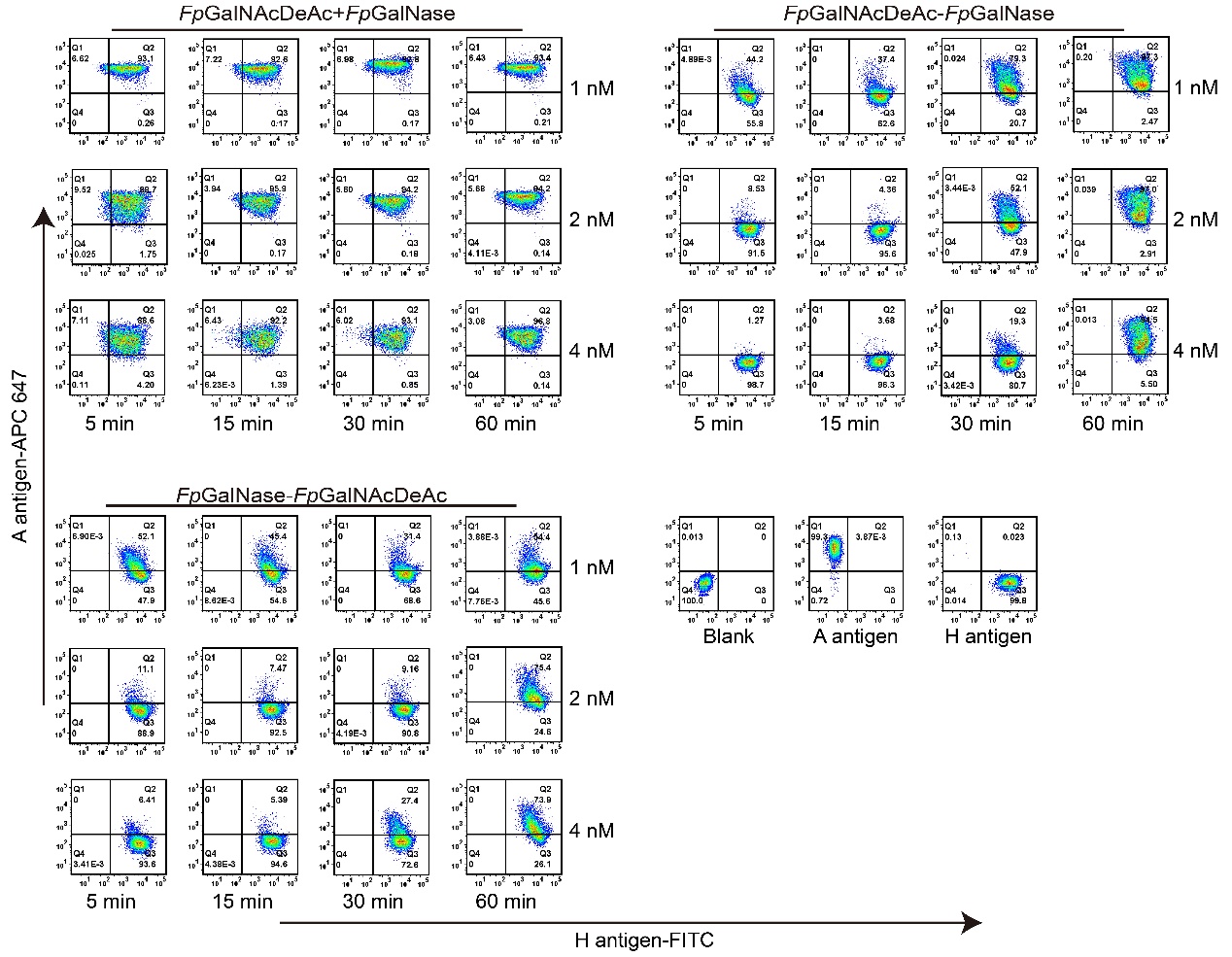


**Fig. S4** FACS analysis showing the relative amounts of A antigen and H antigen on the surface of A-type RBCs treated with the enzyme mixture of *Fp*GalNAcDeAc and *Fp*GalNase, the fusion protein *Fp*GalNAcDeAc-*Fp*GalNase, and the fusion protein *Fp*GalNase-*Fp*GalNAcDeAc. Representative results from three independent experiments are shown.


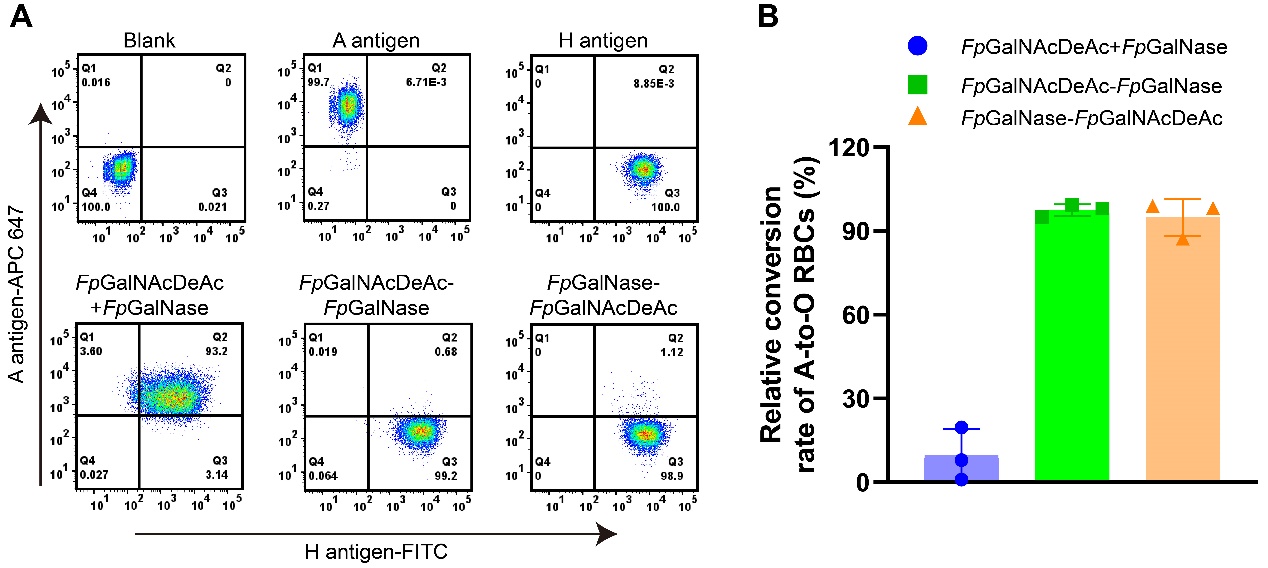


**Fig. S5** FACS analysis (A) and statistical plots (B) showing the relative amounts of A antigen and H antigen on the surface of A-type RBCs treated with 4 nM enzyme mixture of *Fp*GalNAcDeAc and *Fp*GalNase, the fusion protein *Fp*GalNAcDeAc-*Fp*GalNase, and the fusion protein *Fp*GalNase-*Fp*GalNAcDeAc, with a 5-minute incubation. Representative FACS results from three independent experiments are shown. Data are shown as means ± SD from three independent experiments.


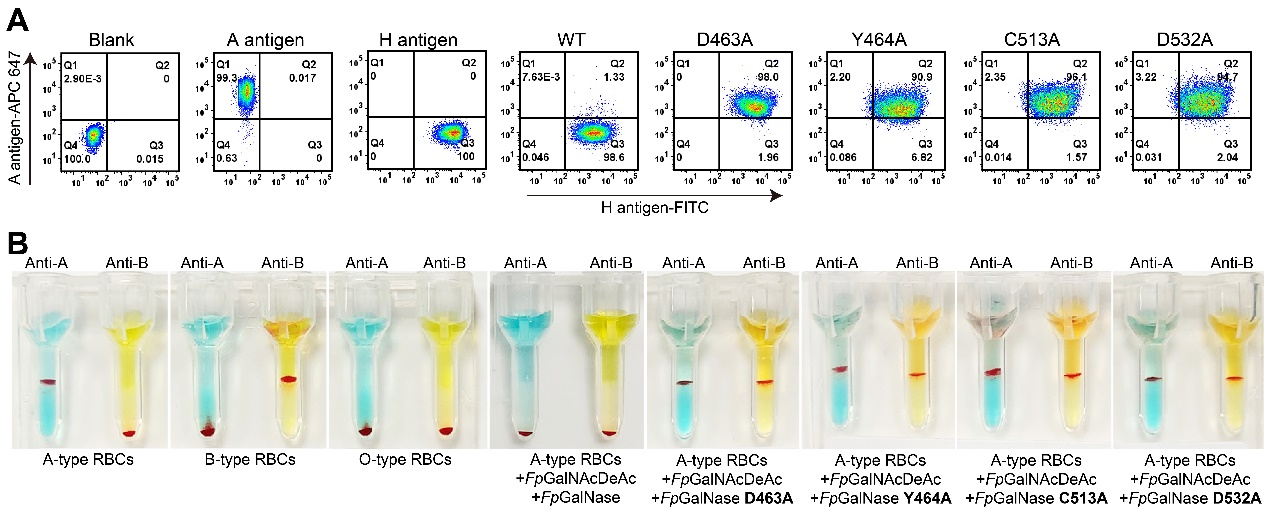


**Fig. S6 Assay of the enzymatic activity of *Fp*GalNase and its mutants.** (**A**) FACS analysis displaying the percentage of A-to-O conversion of A-type RBCs treated with the enzyme mixture of *Fp*GalNase or its mutants and *Fp*GalNAcDeAc. Representative results from three independent experiments are shown. (**B**) Blood group identification of RBCs post-incubation with *Fp*GalNase or its mutants and *Fp*GalNAcDeAc enzymes using a blood group identification card.


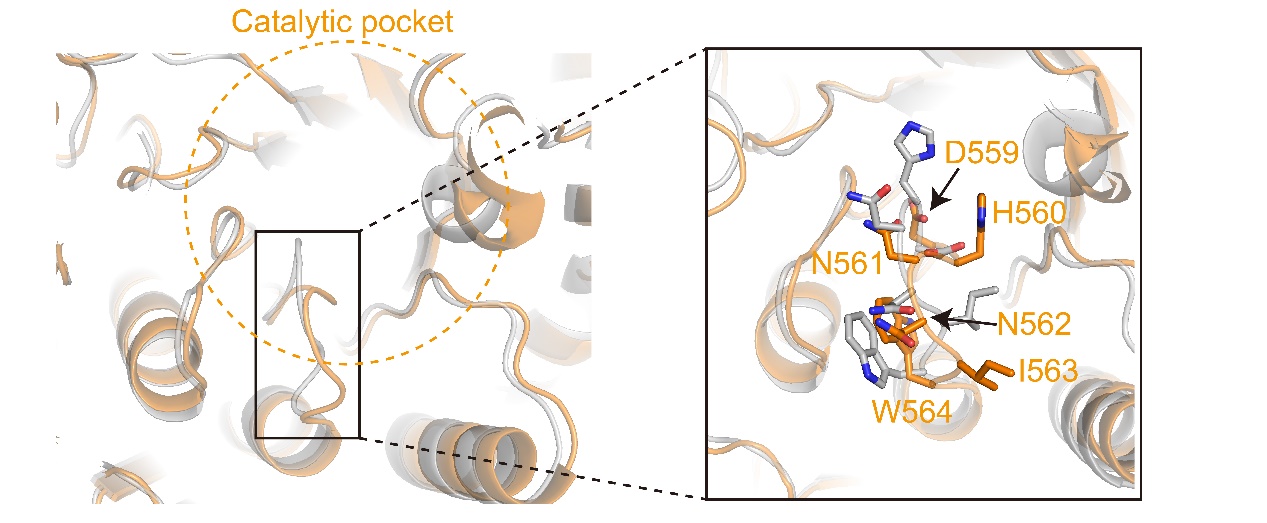


**Fig. S7** Structural alignment of the catalytic pocket between the experimentally resolved (orange) and Alphafold-predicted (gray) *Fp*GalNase. The loop region spanning residues D559 to W564 is highlighted and labeled.

**
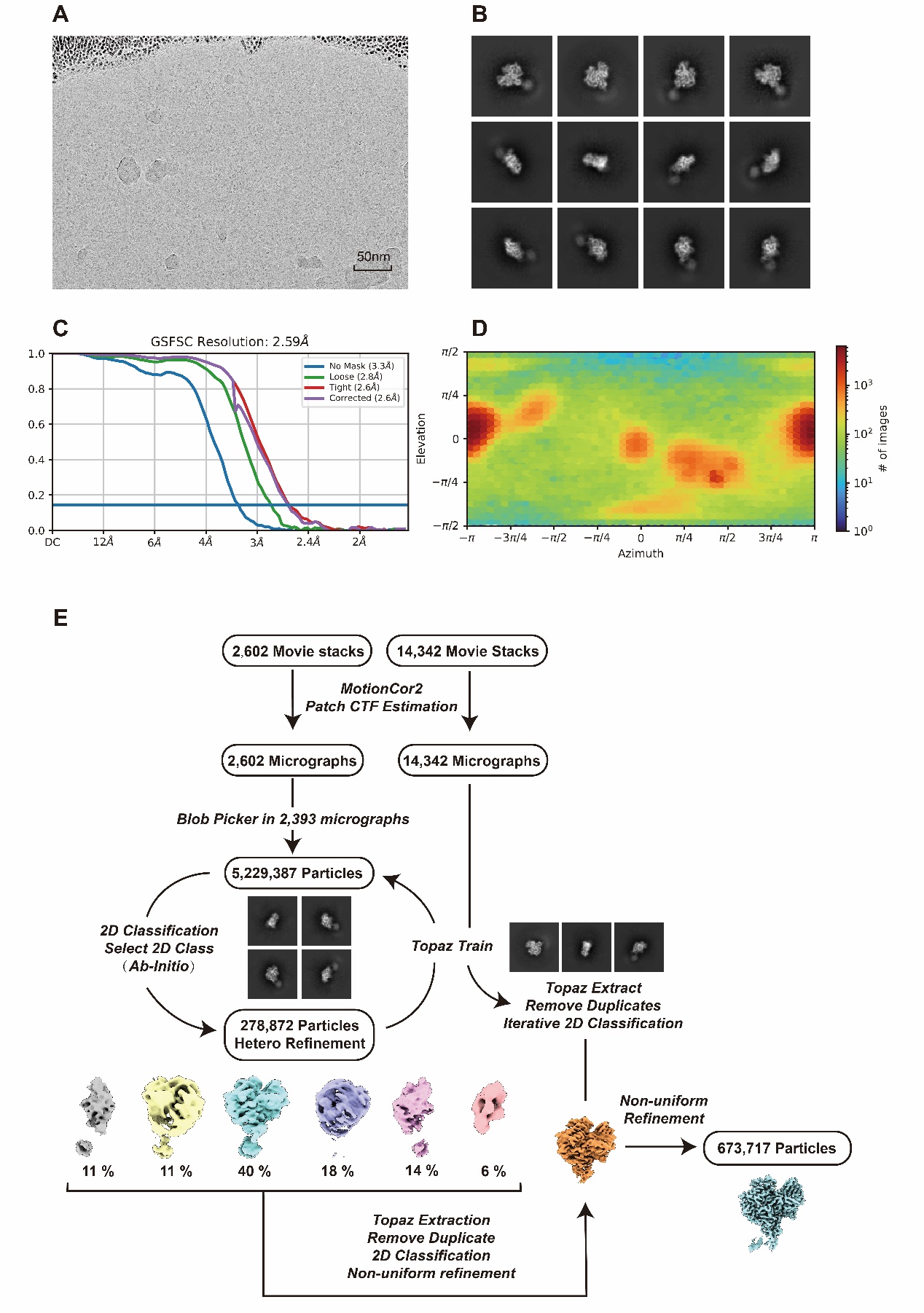
**

**Fig. S8** **The cryo-EM image processing workflow of *Fp*GalNase.** (**A**) A representative cryo-EM micrograph of *Fp*GalNase. Scale bar, 50 nm. (**B**) 2D class average images of *Fp*GalNase. (**C**) The FSC curve for the reconstruction. (**D**) The orientation distribution of the reconstruction. (**E**) A brief workflow of cryo-EM image processing and reconstruction.

**Table S1 Cryo-EM data collection, refinement and validation statistics**

|  | ***Fp*GalNase (PDB: 8X1B)** |
| --- | --- |
| **Data collection and processing** |  |
| Magnificc | 105k |
| Voltage (kV) | 300 |
| Electron exposure (e–/Å^2^) | 50 |
| Defocus range (μm) | -1.0 ~ -2.0 |
| Pixel size (Å) | 0.85 |
| Symmetry imposed | C1 |
| Initial particle images (no.) | 278,872 |
| Final particle images (no.) | 673,717 |
| Map resolution (Å)  FSC threshold | 2.59  0.143 |
|  |  |
| **Refinement** |  |
| Initial model used (PDB code) | P0DTR5 (AlphaFold2) |
| Model resolution range (Å) | 2.59 |
| Map sharpening *B* factor (Å^2^) | 110.8 |
| Model composition  Non-hydrogen atoms  Protein residues | 5106  658 |
| R.m.s. deviations  Bond lengths (Å)  Bond angles (°) | 0.003  0.496 |
| Validation  MolProbity score  Clashscore  Poor rotamers (%) | 1.59  5.35  1.29 |
| Ramachandran plot  Favored (%)  Allowed (%)  Disallowed (%) | 96.65  3.35  0.00 |

**Supplementary references**

1. Zheng SQ, Palovcak E, Armache JP, Verba KA, Cheng Y, Agard DA: **MotionCor2: anisotropic correction of beam-induced motion for improved cryo-electron microscopy**. *Nat Methods* 2017, **14**(4):331-332.

2. Rohou A, Grigorieff N: **CTFFIND4: Fast and accurate defocus estimation from electron micrographs**. *J Struct Biol* 2015, **192**(2):216-221.

3. Punjani A, Rubinstein JL, Fleet DJ, Brubaker MA: **cryoSPARC: algorithms for rapid unsupervised cryo-EM structure determination**. *Nat Methods* 2017, **14**(3):290-296.

4. Pettersen EF, Goddard TD, Huang CC, Couch GS, Greenblatt DM, Meng EC, Ferrin TE: **UCSF Chimera--a visualization system for exploratory research and analysis**. *J Comput Chem* 2004, **25**(13):1605-1612.

5. Emsley P, Cowtan K: **Coot: model-building tools for molecular graphics**. *Acta Crystallogr D Biol Crystallogr* 2004, **60**(Pt 12 Pt 1):2126-2132.

6. Adams PD, Afonine PV, Bunkoczi G, Chen VB, Davis IW, Echols N, Headd JJ, Hung LW, Kapral GJ, Grosse-Kunstleve RW *et al*: **PHENIX: a comprehensive Python-based system for macromolecular structure solution**. *Acta Crystallogr D Biol Crystallogr* 2010, **66**(Pt 2):213-221.
